# The heart rate method for estimating oxygen uptake: analyses of reproducibility using a range of heart rates from commuter walking

**DOI:** 10.1007/s00421-019-04236-0

**Published:** 2019-10-18

**Authors:** Peter Schantz, Jane Salier Eriksson, Hans Rosdahl

**Affiliations:** The Research Unit for Movement, Health and Environment, The Åstrand Laboratory and Laboratory for Applied Sport Science, The Swedish School of Sport and Health Sciences, GIH, Stockholm, Sweden

**Keywords:** Walking commuting, Pedestrians, Heart rate, Oxygen uptake, Heart rate–oxygen uptake relation, Metabolic measurements, Rated perceived exertion, Reproducibility

## Abstract

**Background:**

The heart rate method, based on the linear relation between heart rate and oxygen uptake, is potentially valuable to monitor intensity levels of physical activities. However, this depends not least on its reproducibility under standard conditions. This study aims, therefore, to evaluate the reproducibility of the heart rate method in the laboratory using a range of heart rates associated with walking commuting.

**Methods:**

On two different days, heart rate and oxygen uptake measurements were made during three submaximal (model 1) and a maximal exercise intensity (model 2) on a cycle ergometer in the laboratory. 14 habitual walking commuters participated. The reproducibility, based on the regression equations from test and retest and using three levels of heart rate from the walking commuting, was analyzed. Differences between the two models were also analyzed.

**Results:**

For both models, there were no significant differences between test and retest in the constituents of the regression equations (*y* intercept, slope and *r* value). Neither were there any systematic differences in estimated absolute levels of VO_2_ between test and retest for either model. However, some rather large individual differences were seen in both models. Furthermore, no significant differences were seen between the two models in slopes, intercepts and *r* values of the regression equations or in the estimated VO_2_.

**Conclusion:**

The heart rate method shows good reproducibility on the group level in estimating oxygen consumption from heart rate–oxygen uptake relations in the laboratory, and based on three levels of heart rate which are representative for walking commuting.

## Introduction

To monitor metabolic demands and physiological work intensities of physical activities in free-living field conditions is of great value in both physical and health education, promotion, surveillance and research. For that purpose a number of small, lightweight and portable instruments for indirect calorimetric measurements have been developed. However, they are costly, technically complicated, and can be sensitive to ambient conditions (MacFarlane 2017; Salier Eriksson et al. 2012; Schantz et al. 2018), which makes them difficult to use in a large scale in research as well as in educational contexts. Furthermore, relevant methodological evaluations of them in laboratory (cf. Rosdahl et al. 2010) or in field conditions (Salier Eriksson et al. 2012; Schantz et al. 2018) are rare.

This motivates a renewed interest in the heart rate method (HR method). It is based on a linear relationship between heart rate (HR) and work rate/oxygen uptake (VO_2_) during exercise, as described during the first half of the twentieth century (Boothby 1915; Krogh and Lindhard 1917; Hohwü Christensen 1931; Berggren and Hohwü Christensen 1950). HR recordings from various physical activities have since then been used in numerous studies as a basis for interpreting energy requirements and exercise intensities in humans (e.g., Bradfield 1971; Åstrand 1971; cf. Montoye et al. 1996; Achten and Jeukendrup 2003; Shephard and Aoyagi 2012) as well as in animals (cf. Green 2011). The value of such measurements is greater if individual HR–VO_2_ relations are established (cf. Montoye et al. 1996, p. 103), which today is facilitated by portable heart rate recorders and automatized stationary metabolic measurement devices. Furthermore, the relation between standardized work rates on ergometer cycles and VO_2_ can, due to a small interindividual variability in mechanical efficiency (for 2/3 of both male and female subjects within 6%, according to Åstrand and Rhyming 1954), be used as a substitute for measuring VO_2_ (cf. Åstrand and Ryhming 1954; Åstrand 1960, 1971), if taking into account that body weight affects these energy demands at standardized work rates (Åstrand et al. 1960; Berry et al. 1993; Lafortuna et al. 2008; Björkman 2017). That enables the heart rate method to also be used for purposes such as health education and promotion in which the exact levels of VO_2_ are not necessary to establish. In that way, different physical activities can be related on an individual basis in terms of, e.g., energy demands (kilocalories/-joules) and intensity levels (metabolic equivalents of task), and interpreted in relation to dose of physical activity and effects on, e.g., health outcomes (cf. Paffenbarger et al. 1986; Hu et al. 1999).

However, the mentioned practice of using a method is one thing, validity and reproducibility is another. Already Berggren and Hohwü Christensen (1950) stated that the HR method must be used “with great care” since the HR “can vary independent of metabolic rate.” There are a number of issues related to validity of the HR method, e.g., the external validity of the HR–VO_2_ relations from laboratory to field settings, and to various types of physical activities, as well as with different durations and ambient conditions that need to be studied in their own rights. Here we instead focus on the fundamental need of evaluating the reproducibility of the HR method under controlled laboratory conditions and to, in relation to previous studies, further the methodological approaches used.

Studies have indicated that the HR response to a repeated standardized cycle ergometer work rate may sometimes be stable and sometimes vary (cf. Montoye et al. 1996, p. 101). One reason for non-stability is a habituation effect of varying magnitude, but leading to a lower pulse rate at a given submaximal work rate. To our knowledge, this is described for the first time by Per-Olof Åstrand in his doctoral thesis (Åstrand 1952, p. 20), and the direction of this effect was later clarified by him in an interview (Eriksson and Larsson 2001). As a consequence, Åstrand did not make use of his first test results for the thesis, and neither was the Åstrand–Ryhming ergometer cycle test (Åstrand and Ryhming 1954) based on the first tests, but on the result of the second test (Eriksson and Larsson 2001, p. 17). The habituation effect has later been stated by Åstrand (1976), and noted by Ekblom-Bak et al. (2014). Another reason for instability in the HR response is a non-systematic day to day variability (Berggren and Hohwü Christensen 1950; cf. Montoye et al. 1996; Achten and Jeukendrup 2003). Whereas habituation effects can be handled through pretest trials, a day to day variability is more difficult to circumvent, and can jeopardize the reproducibility of the HR–VO_2_ relation under controlled laboratory conditions. In such case, the extent to which we can rely on measurements in the laboratory for interpretation in field condition will indeed also be hampered.

HR–VO_2_ relations during physical activity in the laboratory can be established through multiple submaximal and maximal work rates, and the pairs of HR and VO_2_ data used to calculate a linear regression equation. It is thereby relevant to evaluate the reproducibility of the HR–VO_2_ method on the basis of the equations, as well as the outcomes of them using different levels of HR to estimate VO_2_. Surprisingly enough, such evaluations in humans, have, to our knowledge, only been the focus of two studies (Christensen et al. 1983; McCrory et al. 1997). Both of them used a single HR level for their evaluations of the outcomes. One of the studies was dominated by patients with different clinical disorders (Christensen et al. 1983). In it, the HR–VO_2_ relation was established from rest to low and intermediate work rates of walking and ergometer cycling. A great variability in the outcomes, based on rather low heart rate values from a 24-h registration, led the authors to conclude that the “applied procedure seems unsuitable for metabolic studies in individual patients who engage in ordinary daily activities with low energy expenditure” (Christensen et al. 1983). McCrory et al. (1997) studied the reproducibility in healthy subjects. Two different HR–VO_2_ relations were established based on measurements from resting to walking. Their single-point HR evaluation was also based on HR recordings from a normal day (ca. 15 h). In the HR–VO_2_ relation which was based on solely walking, a good reproducibility was noted on the group level, whereas a certain variability was noted in the individual levels.

**Fig. 1:**
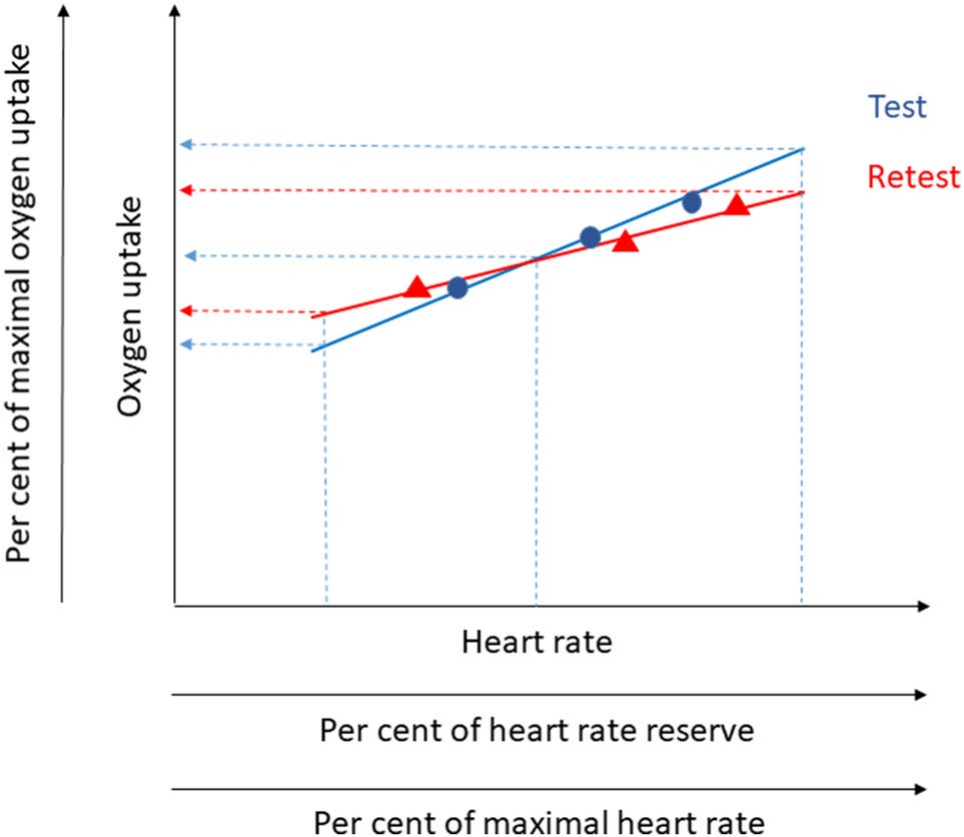
An illustration of how a variability in HR–VO_2_ relations at test and retest can affect measures of reproducibility. Linear relations from regression equations, based on values from three submaximal work rates at test and retest, are illustrated as unbroken lines. Based on different HR and the regression equations, the estimated levels of VO_2_ can be higher, equal or lower at test compared to retest (see broken lines)

The conflicting results, and evaluations based on only one, and rather low levels of HR, together with the paucity of critical evaluations of the HR–VO_2_ method, prompted us to scrutinize these matters further. Methodological issues that need to be addressed relate to the degree of reproducibility possibly varying within one and the same study depending on the levels of HR being used for the evaluations (Fig. 1). If, for example, regression equation slopes from test and retest cross each other, an excellent reproducibility will be attained at that point. However, on both sides of it, the absolute differences in estimated VO_2_ will increase, but in different directions. A great number of other potential interrelations between dual regression slopes and *y* intercepts can produce a substantial variation in the test–retest variability. The magnitude of those differences may, however, be of no importance if they occur outside of the range of HR of interest. The reproducibility of VO_2_ estimations, based on HR–VO_2_ relations, needs, therefore, to be studied at several levels of HR that are distributed along a relevant range of HR.

Another factor that is likely to determine the degree of reproducibility is the number and span of work rates that are used to establish the HR–VO_2_ relations. To enable systematic studies of these matters, it is, therefore, important to specify the levels of HR used in terms of both absolute levels and percentages of maximal heart rate (Londeree and Ames 1976) as well as the heart rate reserve (HRR) (Karvonen et al. 1957; Swain and Leutholtz 1997). The corresponding levels of VO_2_ and their percentage of the maximal oxygen uptake are also of value to describe (Fig. 1).

Walking commuting has been stated to occur at lower heart rates and relative exercise intensities than cycling (Oja et al. 1991) which, given the background above, may affect the reproducibility of the heart rate method. The aim of this study was, therefore, to evaluate day-to-day reproducibility of HR–VO_2_ regression equations and the estimated oxygen uptakes based on three levels of heart rates representative for everyday walking commuting. Two HR–VO_2_ relations were established and compared, one with three levels of submaximal exercise (model 1), and another which also included a maximal exercise (model 2). Model 2 has previously been used in research studies (e.g., Åstrand 1971; Schantz 1980; Schantz et al. 1983). Model 1 has been extensively used in health education at our university college, and has the advantage of not including a maximal exercise test. The HR–VO_2_ relations were attained on an ergometer cycle in the laboratory in healthy and physically active middle-aged male and female walking commuters.

## Methods

### Participants

Approval to conduct the study was obtained from the Ethics Committee North of the Karolinska Institute at the Karolinska Hospital (Dnr 03-637), Stockholm, Sweden.

#### Recruitment of participants

The process of selecting participants was divided into several steps. It started with recruitment through advertisements in two large morning newspapers in Stockholm in May and June of requesting participants. The inclusion criteria required being at least 20 years old; living in the county of Stockholm (excluding the municipality of Norrtälje); walking or cycling the whole way, any distance, between home and to one’s work or place of study, and actively commuting in that fashion at least once a year. Answers could be sent in cost-free by post, fax, e-mail or by phone. These advertisements resulted in 2148 people volunteering to take part.

A questionnaire (The Physically Active Commuting in Greater Stockholm Questionnaire 1; PACS Q1) was sent home to these volunteers; 2010 were returned after three reminders. The questionnaire consisted of 35 questions, but only the questions relevant to selecting our population were used in this study. These included gender, age, how physically strenuous their professional jobs were, commuting frequencies per week for each month of the year and commuting duration. The commuting distance of each individual was also used for selecting the study group. These were measured on routes drawn in maps by each respondent. The mapped route distance measuring method is described in detail in Schantz and Stigell (2009). From the answers from PACS Q1, the respondents were divided into categories based on their reported mode of either cycling or walking, or combining both modes.

Our sample was selected from the pedestrian category, i.e., those subjects who only walked to work. Other criteria were that they had ages and route distances close to the median values of the male and female pedestrians, respectively (cf. Stigell and Schantz 2015). They also rated their daily professional jobs as light or very light physically. Letters describing the physiological studies and test procedures were sent to the male and female pedestrians who fulfilled the criteria.

The recipients of the letter were first asked if their previously drawn route was still valid, or of a comparable distance time wise (comparably defined as plus/min 5–10 min). They then answered a health declaration concerning (1) medication and for which kind of illness, (2) if they had any palpitations, chest pain or abnormally heavy breathing during exercise, (3) if they had high blood pressure, and (4) if they had recently avoided or discontinued exercise for reasons of injury or health. The letter emphasized the right to terminate the tests at any time and without having to stipulate a reason. A signed informed consent of participation was returned.

Based on this information, individuals with invalid route distances as well as with high blood pressure or on medication that could affect normal heart rate were excluded. Anyone on medication with risks for strong side effects was also excluded. We contacted the remaining pedestrians by telephone to answer any potential questions, and to book test times. Telephone contacts continued until we had seven men and seven women who fulfilled the criteria and were willing to participate (Table 1).

**Table 1:** Characteristics of the participants, their walking commuting trips and walking environments (mean ± SD)

Walking commuters	Age (years)	Height (cm)	Weight (kg)	BMI (kg·m^−2^)	Duration (min)	Distance (km)	Velocity (km·h^−1^)	Trips per year	Walking environment^a^
Males (*n* = 7)	48.4 (11)	181 (7)	84 (12)	26 (3)	25.9 (11.4)	2.4 (0.9)	5.7 (0.4)	364 (129)	0.29 (0.76)
Females (*n* = 7)	44 (5)	169 (5)	62 (9)	22 (3)	20.3 (6.2)	2.0 (0.7)	6.0 (0.4)	437 (36)	1.14 (0.9)

^a^Walking environment: 0 = inner urban; 1 = inner urban–suburban; 2 = suburban

### Equipment and preparation

#### Stationary metabolic gas analysis system

A stationary metabolic gas analysis system, the Oxycon Pro^®^ (Carefusion GmbH, Hoechberg, Germany) was used in the mixing chamber mode for all metabolic measurements in the laboratory. The software used was JLAB 4.53. In this system, the concentration of oxygen is measured by a paramagnetic analyzer and the carbon dioxide concentration by an infra-red analyzer. The expired air is sampled continuously from the mixing chamber through a Nafion tubing on the outside of the equipment that connects to a Nafion tubing on the inside of the equipment and that terminates at the analyzer inlets. Ventilation is measured through a digital volume transducer which is attached to the outlet of the mixing chamber. The equipment was switched on 30 min before data collection and calibrated before and after each test using the built-in automated procedures and according to the manufacturer’s recommendations. The ambient conditions were first recorded, followed by calibration of the volume sensor and the gas analyzers. A high-precision gas of 15.00% O_2_ and 6.00% CO_2_ (accuracy O_2_ ± 0.04% rel. and CO_2_ ± 0.1% rel. Air Liquide AB, Kungsängen, Sweden) was used for calibration.

A face mask with non-rebreathing air inlet valves (Combitox, Dräger Safety, Lübeck, Germany) was used. It was carefully fitted on the subject and checked for air leakage immediately prior to the measurements by the investigator and adjusted until no leakage occurred. For several subjects, a rubber insert was taped inside the top of the mask to prevent air leakage from the bridge of the nose. A tube (inner diameter of 35 mm) attached to the mask led the expired air into the mixing chamber. The measured variables were exported to Excel for further processing.

#### Ergometer cycle

A manually braked pendulum ergometer cycle (828E Monark Exercise AB, Vansbro, Sweden) was used. Before each experiment, the scale was zeroed while each subject sat on the saddle with his or her feet resting on the frame between the pedals, and hands resting on the handle bars. The saddle height was adjusted so that the participant’s knees were slightly flexed when the feet were on the pedals in their lowest position. The handle bars were adjusted to allow the participants to sit in an upright position. A digital metronome (DM70 Seiko S-Yard Co. Ltd, Tokyo, Japan) helped the subjects maintain the correct cadence while cycling. The work rate was controlled every minute by checking the cadence of the participant and the braking force as indicated on the pendulum scale.

#### Heart rate

During the resting period and the exercise protocol, HR was measured using a Polar Electro S610i Heart Rate Monitor, with a Polar Wearlink 31 transmitter (Polar Electro Oy, Kempele, Finland).

### Measurements

#### Laboratory tests, standardization procedures and rest conditions

The walking commuters were tested in the laboratory at rest, and submaximal as well as maximal work rates on two different occasions, which were completed within an average of 8.0 ± 4.7 days. Two trained investigators carried out the laboratory tests, each participant having the same investigator for each test. The participants were not able to drink during any of the tests.

The pedestrians were asked to follow the same standard procedures before each test occasion. These were (1) not to engage in any vigorous exercise for 24 h beforehand, (2) not to cycle to the laboratory, (3) to refrain from eating, drinking, smoking and taking snuff (smokeless tobacco) for at least 1 h before arrival at the laboratory, (4) not to eat a large meal at least 3 h before the tests, (5) to avoid stress and (6) to cancel the test if they had fever, an infection or a cold. The time of the day that the tests were undertaken was not standardized since it does not affect the HR–VO_2_ relation during physical activity (McCrory et al. 1997). The participants wore light clothes, such as T-shirts, shorts and training shoes, so as to diminish any effect of the energy liberation from the submaximal exercises on sweating and body temperature.

On arrival at the laboratory, the participants were weighed and measured, and a check list was ticked off to determine if they had followed the standard procedures named above. A Polar heart rate monitor and a Wearlink were then placed on a wrist and around the chest, respectively. The participants rested quietly for 10 min on a treatment table. Resting heart rate, calculated from the time period between every single heart rate, was determined from the average of the 5 min between the 6th and 10th min.

#### Cycle ergometer exercise protocol

The participants cycled at three different work rates: 50, 100 and 150 W for the women, and 100, 150 and 200 W for the men. A cadence of 50 revolutions per minute was chosen (Åstrand 1952, p. 19). At each work rate, the participant cycled until steady state (approximately 6 min), after which the resistance was increased. The third work rate was increased to only 125 W or 175 W for women and men, respectively, if, after the second work rate, the subject’s HR was higher than 150 beats per minute and their perceived rate of exertion exceeded 15 for both legs and breathing (Borg 1998, p. 30). The HR from the Polar heart rate monitor, with the HR averaged for every 15 s, and RPE was noted in the protocol after every minute.

Between the second and third work rates, the test person continued cycling for 1 min at a self-chosen low cadence with a resistance of 5 N. The subject was then instructed to resume the cadence of 50 rpm while the investigator slowly increased the work rate until, after 1 min, the third work rate was reached. For that purpose, resistance was increased to 50 W during the first 15 s, to 100 W the second 15 s and successively to the required work rate during the last 30 s. After the third submaximal test, the subject continued cycling for 2 min at a self-chosen low cadence at 5 N.

During the maximal exercise phase, the subjects cycled at a cadence of 80 rpm (Foss and Hallén 2004). For the first three minutes, the work rates were 60, 100, and 120 or 140 W for 1 min each. The latter alternatives depended on which third work rate the subjects had during the submaximal work: 120 W if the third submaximal work rate had been 125 W or 175 W for women and men, respectively; 140 W if it had been 150 W or 200 W for women and men, respectively. The work rate increased thereafter by 20 W every 60 s. The test continued until exhaustion. HR was noted before each increase of the resistance and also at the moment when the participant terminated the test because of exhaustion.

To assess the rating of perceived exertion (RPE), the Borg scale, as mentioned before, was used (Borg 1998, p.30). The subjects were instructed on how to use the scale before commencing the tests. They were asked to point to a number on the scale that corresponded to their feeling of exertion for breathing and in their legs, respectively, before every increase of resistance during the submaximal test and directly after the maximal test. During the maximal phase they continued until exhaustion. To ensure that each subject achieved maximal exertion, at least two of the following three criteria were met by each subject: (1) a plateau in VO_2_ despite increasing exercise intensity (defined as a VO2 increment of less than 150 ml), (2) a respiratory exchange ratio of ≥ 1.1, and (3) a rating of RPE of ≥ 17 on the Borg scale (Borg 1970; Howley et al. 1995; Midgley et al. 2007).

The laboratory tests were completed within an average of 18.9 ± 17.7 days (23.7 ± 21.5 days for men; 14.1 ± 12.2 days for women).

#### Measurements of heart rate during commuter walking

The participants commuted either to or from their workplace choosing themselves which time was most convenient. 18 of the pedestrians (nine men; nine women) were tested in the morning (start times between 07:26 h and 10:15 h) and the remaining two women were tested after work (start times 16:47 h and 16:48 h). The field trips took place in the inner urban and suburban–rural areas of Stockholm, Sweden. A detailed description of these areas can be found in Wahlgren and Schantz (2011). The majority of the pedestrians walked at least partly within the inner urban area (cf. Table 1). They were met at the designated address by one of the investigators, who checked that the pretest standardization procedures, as described above, had been followed. The participants were instructed to walk at their normal pace. HR was measured using the Polar electro heart rate recorder and the HR was averaged for every 15 s. The starting time of the walking trip was synchronized with the second investigator waiting at the destination. The participants were not able to drink during their walk commute.

On arrival at the destination, the total trip time was noted and the walker was asked to rate his perceived rate of exertion for both breathing and legs. They were asked how many stops that were made at traffic lights as well as other stops, and marked them on maps with their routes. The participants were also asked to confirm whether that route had been taken the whole way, and if not, any deviation from the originally marked route was added to the map.

### Analytical approach and statistical analyses

For determining the resting HR, the values are based on each single time period between the heart beats, which were transformed into heart rates per minute and averaged for a 5-min period.

For the submaximal tests in the laboratory, the mean of the four 15-s values for VO_2_ and HR for the last minute of each load was used for analysis. The values for the maximal tests were calculated by averaging the highest four 15-s consecutive values for VO_2_ and HR at maximal exercise, i.e., a collection period of 60 s (Howley et al. 1995). The same corresponding values were used for both VO_2_ and HR.

The reproducibility of the paired individual data for VO_2_ and HR between test and retest in the laboratory was calculated as absolute and relative differences, and analyzed with Wilcoxon’s signed rank test and Student’s paired *t* test as well as coefficient of variation (CV). The CV was calculated by dividing the standard deviation of the difference between the test–retest values by √2. This value (typical error) was then divided with the average of the test–retest values and multiplied by 100 (Hopkins 2000). With regard to significance, the same results were obtained with the non-parametric Wilcoxon’s signed rank test and the parametric Student’s paired *t* test. In the further statements with regard to analyses undertaken, and in “Results”, we do, however, only report the values obtained from the Student’s paired *t* test.

The HR–VO2 relations based on each individual´s paired VO_2_ and HR from three submaximal work rates (model 1) plus a maximal work rate (model 2) at test and retest were described by linear regression analyses and correlation coefficients (*r* values). The absolute differences in *y* intercepts, slopes and *r* values between test and retest were evaluated with paired Student’s *t* test for each model. The absolute values for the *y* intercepts, slopes and *r* values at test and retest were also compared between models 1 and 2 with paired Student’s *t* test, and the 95% confidence intervals for the mean values were also calculated.

The reproducibility of the estimated VO_2_, based on the regression equations from test and retest, and calculated on the basis of three levels of HR from each individual’s cycle commuting, was calculated as absolute and relative differences. They were analyzed for all individuals with Student’s paired *t* test, the 95% confidence intervals for the mean values and coefficient of variation (CV).

Whether the levels of estimated VO_2_ at test and retest, as well as the differences between test and retest, were altered between models 1 and 2 was also evaluated with Student’s paired *t* test and 95% confidence intervals for the mean values. Bland–Altman plots with 95% limits of agreement in individual absolute values of estimated VO_2_ were graphically displayed (Bland and Altman 1986).

Statistical analyses were performed using the Statistical Package for the Social Sciences (SPSS, 17.0, Chicago, IL, USA). The Bland–Altman plots were created with Graph-Pad Prism^®^ 4 software package (Graph-Pad Software Inc., San Diego, CA, USA). Values are presented as mean ± SD unless otherwise stated. The significance level was set at *p* < 0.05 when data were used only once, and when data were used twice, a Bonferroni correction of the level of significance was done resulting in *p* < 0.025.

## Results

### Reproducibility of repeated single measurements

There were no systematic absolute or relative differences in VO_2_ and HR between the first and second measurement occasions in the laboratory (Table 2).

**Table 2:** Test–retest of VO_2_ and HR at rest, submaximal and maximal cycle ergometer tests in the laboratory (mean ± SD) and coefficient of variation (CV)

	Males (*n* = 7)								Females (*n* = 7)						
	Test	Retest	Absolute difference, L min^−1^	Relative difference, %	*p* values abs	*p* values %	CV		Test	Retest	Absolute difference, L·min^−1^	Relative difference, %	*p* values abs	*p* values %	CV
VO_2_, L min^−1^															
100 W	1.53 ± 0.15	1.53 ± 0.08	0.00 ± 0.12	0.6 ± 7.7	1.000	0.851	5.4	50 W	0.83 ± 0.03	0.83 ± 0.06	0.00 ± 0.06	0.6 ± 7.8	0.864	0.844	5.4
150 W	2.10 ± 0.26	2.11 ± 0.30	0.01 ± 0.13	0.4 ± 6.9	0.843	0.885	4.3	100 W	1.44 ± 0.07	1.42 ± 0.05	− 0.03 ± 0.10	− 1.7 ± 6.8	0.485	0.528	5.0
168 ± 19 W	2.45 ± 0.26	2.43 ± 0.27	− 0.03 ± 0.14	− 1.1 ± 6.0	0.627	0.660	4.1	132 ± 12 W	1.85 ± 0.16	1.84 ± 0.19	− 0.1 ± 0.08	− 0.5 ± 4.4	0.784	0.760	3.0
Maximal	2.96 ± 0.48	3.10 ± 0.49	0.14 ± 0.16	4.7 ± 5.8	0.064	0.072	3.7	Maximal	2.33 ± 0.26	2.31 ± 0.23	− 0.02 ± 0.14	− 0.4 ± 6.5	0.784	0.884	4.4
HR beats, min^−1^															
Rest	71 ± 7	73 ± 6	2.14 ± 5.4	3.4 ± 7.3	0.332	0.262	5.3	Rest	64 ± 9	62 ± 9	− 2.43 ± 6.0	− 3.5 ± 9.6	0.323	0.365	6.7
100 W	117 ± 15	115 ± 13	− 2.14 ± 8.49	− 1.5 ± 6.5	0.529	0.574	5.2	50 W	98 ± 9	97 ± 7	− 1.14 ± 4.60	− 1.0 ± 4.6	0.535	0.600	3.3
150 W	141 ± 15	137 ± 12	− 4.14 ± 13.9	− 2.4 ± 9.0	0.460	0.504	7.1	100 W	130 ± 11	129 ± 11	− 0.14 ± 4.95	0.0 ± 3.8	0.942	0.988	2.7
168 ± 19 W	157 ± 11	152 ± 11	− 4.86 ± 11.1	− 2.9 ± 6.6	0.291	0.292	5.1	132 ± 12 W	154 ± 7	152 ± 8	− 2.00 ± 3.27	− 1.3 ± 2.2	0.156	0.163	1.5
Maximal	180 ± 10	181 ± 12	0.71 ± 5.47	0.4 ± 3.2	0.741	0.771	2.1	Maximal	176 ± 9	177 ± 8	1.00 ± 4.16	0.6 ± 2.5	0.549	0.535	1.7

The *p* values are based on the paired differences in absolute values and percent differences

### Positioning work rates for the HR–VO_2_ relations in the laboratory

The three submaximal work rates, used in both models of HR–VO_2_ regression equations, induced mean levels of HR ranging from on average 117 ± 15 to 157 ± 11 beats per minute for the males, and from 98 ± 9 to 154 ± 7 for the females (Table 2). For maximal HR and other descriptive aspects of the work rates used, see Tables 2 and 3.

**Table 3:** Positions of the ergometer cycle work rates used to determine the HR–VO_2_ relations in males and females and expressed as per cent of VO_2max_, heart rate reserve and HR_max_, as well as rated RPE during test 1 (mean ± SD)

	Work rates on an ergometer cycle in the laboratory							
Sex	Males (*n* = 7)				Females (*n* = 7)			
Work rates	100 W	150 W	168 ± 19 W	Maximal	50 W	100 W	132 ± 12 W	Maximal
Percent of maximal oxygen uptake	52.6 ± 8.0	71.6 ± 7.1	83.7 ± 9.0	100	35.8 ± 3.8	62.6 ± 7.5	79.7 ± 4.1	100
Percent of heart rate reserve	43.0 ± 12.5	65.0 ± 14.5	78.9 ± 12.6	0	29.5 ± 7.2	57.9 ± 9.8	80.0 ± 5.8	0
Percent of maximal heart rate	65.3 ± 8.3	78.6 ± 8.9	87.1 ± 7.6	100	55.4 ± 3.7	73.5 ± 5.2	87.5 ± 2.9	100
Rated perceived exertion, legs	11.7 ± 2.0	14.3 ± 1.4	15.7 ± 1.0	18.2 ± 0.8	10.1 ± 1.9	13.1 ± 1.2	15.6 ± 1.1	18.0 ± 1.3
Rated perceived exertion, breathing	11.4 ± 1.6	13.6 ± 2.2	14.9 ± 2.3	17.0 ± 2.3	9.7 ± 1.3	12.6 ± 0.8	15.1 ± 2.0	18.2 ± 1.7

### Positioning the HR from the commuter walking used for estimating levels of VO_2_

The mean values of the lowest, middle and highest fifth of heart rates during each participants’ walking commute were used to estimate the corresponding level of VO_2_ based on the HR–VO_2_ regression equations. These heart rate segments were determined through ordering all heart rates from the lowest to the highest, and then dividing them into segments of 1/5 of all heart rates. It is the segments with the 20% lowest, intermediate and highest heart rates that are described in Table 4. The range of mean HR levels was between 104 ± 17 and 124 ± 20 beats per minute for the males, and from 103 ± 8 to 128 ± 10 for the females. For further details on the responses to the work rates used, see Table 4.

**Table 4:** The three HR levels from walking commuting used to estimate VO_2_ based on the HR–VO_2_ regression equations at test and retest

	Heart rates during walking commuting					
Sex	Males (*n* = 7)			Females (*n* = 7)		
HR segments, %	0–20	41–60	81–100	0–20	41–60	81–100
Heart rate, beats/minute	104 ± 17	116 ± 21	124 ± 20	103 ± 8	114 ± 9	128 ± 10
Percent of heart rate reserve	27.8 ± 13.1	38.8 ± 15.1	46.7 ± 14.3	34.5 ± 8.3	44.3 ± 7.9	56.5 ± 11.2
Percent of maximal heart rate	56.8 ± 9.0	63.4 ± 10.2	68.0 ± 9.6	57.8 ± 3.5	64.0 ± 4.3	72.0 ± 6.5

The corresponding levels of percent of HR_max_ as well as percent of heart rate reserve are also given (mean ± SD)

### Reproducibility of HR–VO_2_ regression equations and estimated levels of VO_2_ (model 1)

**Fig. 2:**
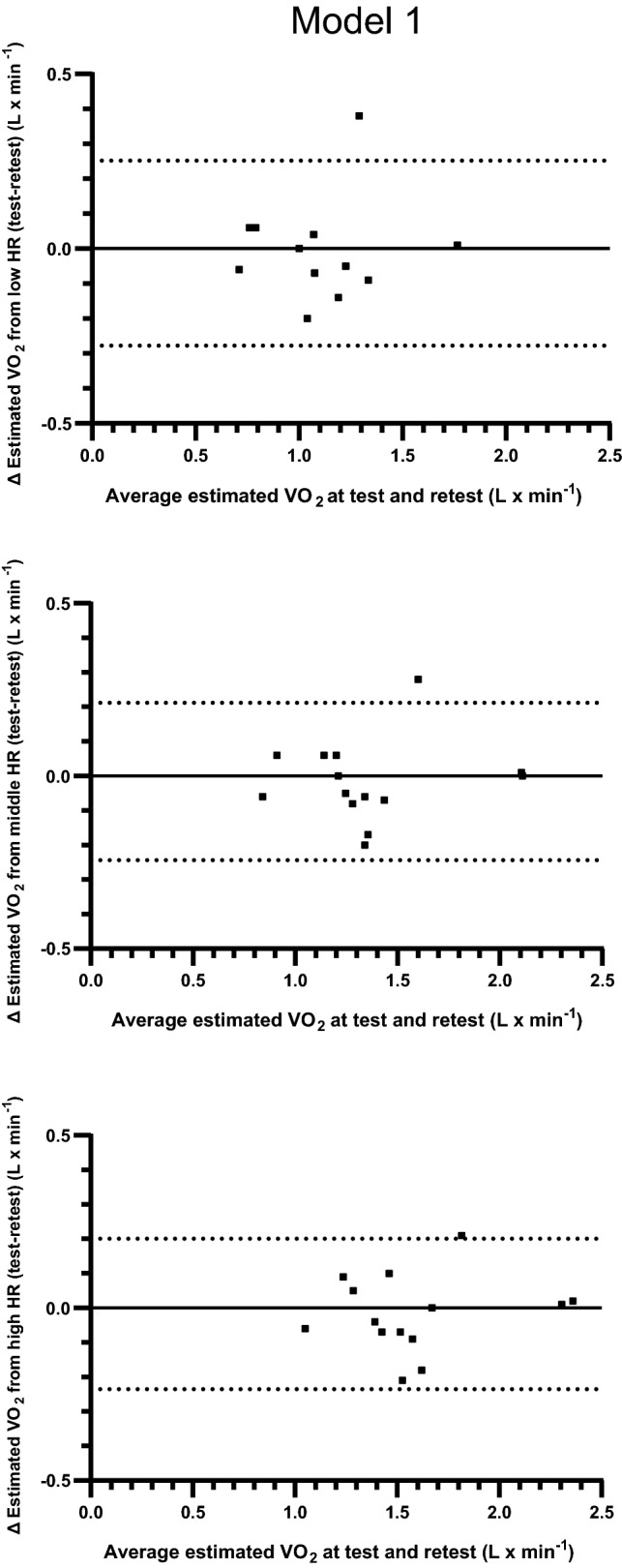
Individual levels of differences and the 95% limits of agreement between estimations of VO_2_ (L · min^−1^) calculated from fixed levels of low, middle and high HR from commuter walking as well as from repeated measurements of VO_2_–HR relations based on three submaximal work rates (Model 1). The *y* axes show absolute differences in VO_2_ against the mean values of the estimations from the repeated measurements on the *x* axes

The test and retest HR–VO_2_ regression equations and estimated levels of oxygen uptake from three levels of HR are presented in Tables 5 and 6. There were no indications of differences in *y* intercept, slope or *r* value in the regression equations at the retest compared to the test (Table 5). Based on calculations of all subjects, there were no systematic differences in estimated absolute levels of VO_2_ between test and retest (Table 6). The relative differences between test and retest were 1.91 ± 11.1, 1.71 ± 8.36 and 1.57 ± 7.34% (all n.s.) based on estimations from the lowest to the highest levels of HR. The 95% limits of agreement for the individual variations in the differences in estimated VO_2_ between test and retest varied between − 0.2775 and 0.2518 (L min^−1^) for the low HR, − 0.2436 and 0.2122 for the middle HR, and − 0.2352 and 0.2008 for the high HR (Fig. 2).

**Table 5:** Reproducibility of HR–VO_2_ regression equations based on three submaximal work rates (model 1) (means ± SD)

Participant	Regression equations					
	Day 1			Day 2		
	*y* intercept	Slope	*r*	*y* intercept	Slope	*r*
Males						
1	− 1.17	0.0235	1.000	− 0.91	0.0229	0.999
2	− 1.26	0.0277	0.979	− 2.65	0.0380	0.979
3	− 0.05	0.0114	0.968	− 0.16	0.0128	0.971
4	− 1.18	0.0241	1.000	− 0.90	0.0207	0.998
5	− 1.90	0.0279	1.000	− 1.50	0.0252	0.989
6	− 0.43	0.0184	1.000	− 0.65	0.0227	1.000
7	− 3.37	0.0383	0.999	− 3.29	0.0376	0.988
Mean	− 1.34	0.0245	0.992	− 1.44	0.0257	0.989
SD	1.08	0.0084	0.013	1.13	0.0091	0.011
Females						
1	− 1.17	0.0221	0.995	− 1.47	0.0254	1.000
2	− 1.08	0.0181	0.996	− 1.03	0.0182	1.000
3	− 0.91	0.0163	1.000	− 0.65	0.0147	1.000
4	− 0.91	0.0191	1.000	− 0.84	0.0191	0.996
5	− 0.77	0.0160	0.999	− 0.73	0.0150	0.998
6	− 1.48	0.0226	0.995	− 1.58	0.0229	0.991
7	− 0.39	0.0144	0.990	− 0.39	0.0144	0.999
Mean	− 0.96	0.0184	0.996	− 0.95	0.0185	0.998
SD	0.34	0.0031	0.003	0.43	0.0043	0.003
All						
Mean	− 1.15	0.0214	0.994	− 1.20	0.0221	0.993
SD	0.79	0.0069	0.010	0.86	0.0078	0.009
*P* value for difference between days 1 and 2				0.683	0.465	0.541

**Table 6:** The estimated levels of VO_2_ based on the regression equations from Table 5 (model 1) and three levels of HR from walking commuting (means ± SD), coefficients of variation (CV), and the 95% confidence intervals (CI) for all values

Participant	Average of the lowest, middle and highest fifth of HR at field, and estimations of VO_2_ based on these levels of HR, and the HR–VO_2_ regression equations at day 1 and day 2														
	Lowest fifth of HR					Middle fifth of HR					Highest fifth of HR				
	HR F1	VO_2_:1	VO_2_:2	Abs diff	% diff	HR F3	VO_2_:1	VO_2_:2	Abs diff	% diff	HR F5	VO_2_:1	VO_2_:2	Abs diff	% diff
Males															
1	90	0.94	1.14	0.20	21.5	102	1.24	1.44	0.20	15.7	115	1.53	1.71	0.19	12.30
2	99	1.48	1.10	− 0.38	− 25.4	108	1.74	1.46	− 0.28	− 16.0	115	1.92	1.71	− 0.21	− 11.03
3	110	1.20	1.25	0.05	3.78	119	1.31	1.37	0.06	4.50	126	1.39	1.46	0.07	4.95
4	94	1.09	1.05	− 0.04	− 3.65	100	1.23	1.17	− 0.06	− 4.83	112	1.51	1.41	− 0.10	− 6.54
5	114	1.29	1.38	0.09	6.68	143	2.11	2.11	0.01	0.35	151	2.31	2.30	− 0.01	− 0.54
6	84	1.12	1.26	0.14	12.6	92	1.27	1.44	0.18	13.9	100	1.42	1.63	0.21	14.90
7	134	1.77	1.76	− 0.01	− 0.48	143	2.11	2.10	− 0.01	− 0.70	150	2.37	2.35	− 0.02	− 0.83
Mean	104	1.27	1.28	0.01	2.15	116	1.57	1.58	0.01	1.85	124	1.78	1.80	0.02	1.89
SD	17.2	0.28	0.24	0.19	14.8	20.7	0.41	0.37	0.16	10.9	19.5	0.42	0.38	0.15	9.47
CV				10.5					7.14					5.99	
Females															
1	107	1.20	1.25	0.05	3.96	116	1.40	1.47	0.08	5.50	122	1.53	1.62	0.10	6.29
2	97	0.68	0.74	0.06	8.84	104	0.81	0.87	0.06	7.50	116	1.02	1.08	0.06	6.06
3	119	1.04	1.11	0.07	6.60	130	1.22	1.27	0.05	4.16	140	1.37	1.41	0.04	2.58
4	102	1.04	1.11	0.07	6.95	113	1.24	1.32	0.07	5.80	125	1.48	1.55	0.07	4.88
5	97	0.79	0.73	− 0.06	− 7.13	107	0.94	0.88	− 0.07	− 6.99	128	1.28	1.19	− 0.09	− 6.79
6	102	0.82	0.76	− 0.06	− 7.58	117	1.17	1.11	− 0.06	− 4.96	124	1.31	1.26	− 0.06	− 4.25
7	97	1.00	1.00	0.00	0.05	111	1.21	1.21	0.00	0.04	143	1.67	1.67	0.00	0.03
Mean	103	0.94	0.96	0.02	1.67	114	1.14	1.16	0.02	1.58	128	1.38	1.40	0.02	1.26
SD	8.2	0.18	0.21	0.06	6.76	8.6	0.20	0.23	0.06	5.68	10.0	0.21	0.23	0.07	5.16
CV				4.35					3.75					3.48	
All															
Mean	103	1.10	1.12	0.01	1.91	115	1.36	1.37	0.02	1.71	126	1.58	1.60	0.02	1.57
SD	12.9	0.28	0.27	0.13	11.1	15.2	0.38	0.37	0.12	8.36	15.0	0.38	0.37	0.11	7.34
* p*-value				.723	.529				.618	.457				.566	.437
CI lower				− 0.06	− 4.47				− 0.05	− 3.12				− 0.05	− 2.66
CI upper				0.09	8.29				0.08	6.54				0.08	5.81
CV				8.54					6.01					5.02

### Reproducibility of HR–VO_2_ regression equations and estimated levels of VO_2_ (model 2)

**Fig. 3:**
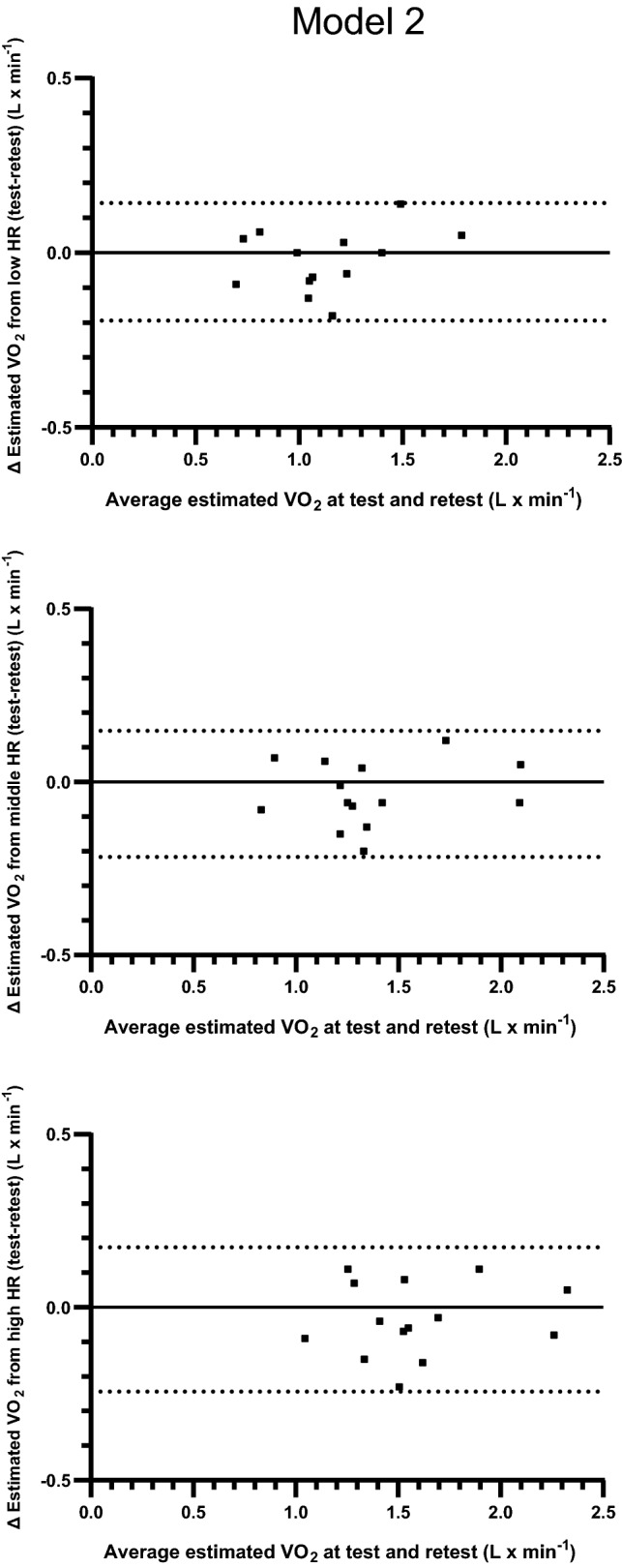
Individual levels of differences and the 95% limits of agreement between estimations of VO_2_ (L · min^−1^) calculated from fixed levels of low, middle and high HR from commuter walking as well as from repeated measurements of VO_2_–HR relations based on three submaximal work rates (Model 2). The *y* axes show absolute differences in VO_2_ against the mean values of the estimations from the repeated measurements on the *x* axes

The test and retest HR–VO_2_ regression equations and estimated levels of VO_2_ from three levels of HR are presented in Tables 7 and 8. There were no significant differences between test and retest in the constituents of the regression equations (*y* intercept, slope and *r* value) (Table 7). Based on calculations of all subjects, there were no systematic differences in estimated absolute levels of VO_2_ between test and retest. The relative differences between test and retest, based on estimations from three different levels of HR, were 3.12 ± 8.25, 3.13 ± 7.60 and 2.81 ± 7.47% (all n.s.) (Table 8). The 95% limits of agreement for the individual variations in the differences in estimated VO_2_ between test and retest varied between − 0.1940 and 0.1428) (L · min^−1^) for the low HR, − 0.2163 and 0.1478 for the middle HR, and − 0.2437 and 0.1737 for the high HR (Fig. 3).

**Table 7:** Reproducibility of HR–VO_2_ regression equations based on three submaximal and a maximal work rate (model 2) (means ± SD)

Participant	Regression equations					
	Day 1			Day 2		
	*y* intercept	Slope	*r*	*y* intercept	Slope	*r*
Males						
1	− 0.93	0.0216	0.998	− 1.10	0.0245	0.999
2	− 0.84	0.0243	0.993	− 1.17	0.0262	0.981
3	− 0.83	0.0165	0.885	− 0.85	0.0179	0.984
4	− 0.61	0.0195	0.970	− 0.42	0.0171	0.967
5	− 1.14	0.0223	0.989	− 1.41	0.0246	0.997
6	− 0.59	0.0197	0.998	− 0.69	0.0230	1.000
7	− 2.78	0.0342	0.996	− 2.80	0.0340	0.994
Mean	− 1.10	0.0226	0.976	− 1.21	0.0239	0.989
SD	0.76	0.0057	0.041	0.78	0.0056	0.012
Females						
1	− 1.08	0.0213	0.998	− 1.03	0.0214	0.991
2	− 1.14	0.0185	0.997	− 1.01	0.0181	1.000
3	− 1.02	0.0172	0.999	− 0.80	0.0159	0.997
4	− 0.98	0.0197	0.999	− 0.95	0.0201	0.997
5	− 1.03	0.0183	0.992	− 0.85	0.0160	0.997
6	− 1.41	0.0220	0.998	− 1.43	0.0217	0.996
7	− 0.42	0.0146	0.994	− 0.49	0.0153	0.997
Mean	− 1.01	0.0188	0.997	− 0.94	0.0184	0.997
SD	0.30	0.0025	0.003	0.29	0.0027	0.003
All						
Mean	− 1.06	0.0207	0.986	− 1.07	0.0211	0.993
SD	0.56	0.0047	0.030	0.58	0.0051	0.009
* p* value diff between days 1 and 2				.751	.376	.386

**Table 8:** The estimated levels of VO_2_ based on the regression equations from Table 7 (model 2) and three levels of HR from walking commuting (means ± SD), coefficients of variation (CV), and the 95% confidence intervals (CI) for all values

Participant	Average of the lowest, middle and highest fifth of HR at field, and estimations of VO_2_ based on these levels of HR and regression Eqs. 1 and 2														
	Lowest fifth of HR					Middle fifth of HR					Highest fifth of HR				
	HR F1	VO_2_:1	VO_2_:2	Abs diff	% diff	HR F3	VO_2_:1	VO_2_:2	Abs diff	% diff	HR F5	VO_2_:1	VO_2_:2	Abs diff	% diff
Males															
1	90	1.01	1.09	0.09	8.74	102	1.28	1.41	0.12	9.75	115	1.54	1.70	0.16	10.4
2	99	1.56	1.42	− 0.14	− 9.04	108	1.79	1.67	− 0.12	− 6.87	115	1.95	1.84	− 0.11	− 5.69
3	110	0.98	1.11	0.14	13.8	119	1.14	1.29	0.15	13.1	126	1.26	1.41	0.16	12.7
4	94	1.23	1.20	− 0.03	− 2.75	100	1.34	1.30	− 0.05	− 3.55	112	1.57	1.49	− 0.08	− 4.80
5	114	1.40	1.40	− 0.01	− 0.50	143	2.06	2.12	0.06	2.93	151	2.22	2.30	0.08	3.47
6	84	1.07	1.25	0.18	16.5	92	1.23	1.43	0.20	16.5	100	1.39	1.62	0.23	16.5
7	134	1.81	1.76	− 0.05	− 2.57	143	2.12	2.07	− 0.05	− 2.28	150	2.35	2.30	− 0.05	− 2.12
Mean	104	1.29	1.32	0.02	3.45	116	1.57	1.61	0.05	4.22	124	1.75	1.81	0.06	4.35
SD	17.2	0.31	0.23	0.11	9.59	20.7	0.41	0.35	0.12	9.01	19.5	0.42	0.36	0.13	8.95
CV				6.10					5.39					5.34	
Females															
1	107	1.20	1.26	0.06	4.74	116	1.39	1.45	0.06	4.16	122	1.52	1.58	0.06	3.86
2	97	0.65	0.74	0.09	14.1	104	0.79	0.87	0.09	11.3	116	1.00	1.09	0.08	8.43
3	119	1.03	1.10	0.07	6.58	130	1.22	1.28	0.05	4.38	140	1.39	1.43	0.04	2.97
4	102	1.03	1.10	0.07	6.61	113	1.24	1.31	0.07	5.83	125	1.49	1.56	0.08	5.21
5	97	0.75	0.71	− 0.04	− 5.86	107	0.93	0.86	− 0.07	− 7.15	128	1.31	1.20	− 0.11	− 8.73
6	102	0.84	0.78	− 0.05	− 6.56	117	1.17	1.11	− 0.06	− 5.08	124	1.32	1.25	− 0.06	− 4.67
7	97	0.99	0.99	0.00	− 0.09	111	1.21	1.22	0.01	0.79	143	1.68	1.71	0.03	1.91
Mean	103	0.93	0.96	0.03	2.79	114	1.14	1.16	0.02	2.04	128	1.39	1.40	0.02	1.28
SD	8.2	0.19	0.21	0.06	7.42	8.6	0.21	0.22	0.06	6.42	10.0	0.21	0.23	0.08	5.95
CV				4.44					3.89					3.83	
All															
Mean	103	1.11	1.14	0.03	3.12	115	1.35	1.39	0.03	3.13	126	1.57	1.61	0.04	2.81
SD	12.9	0.31	0.29	0.09	8.25	15.2	0.38	0.37	0.09	7.60	15.0	0.37	0.36	0.11	7.47
*P* value				.291	.180				.198	.147				.226	.182
CI lower				− 0.02	− 1.64				− 0.02	− 1.26				− 0.03	− 1.50
CI upper				0.08	7.88				0.09	7.52				0.10	7.13
CV				5.44					4.84					4.75

### Comparisons of regression equations and estimated VO_2_ between the HR–VO_2_ relations in models 1 and 2

The differences between the two HR–VO_2_ models in the *y* intercept, slope, *r* value as well as in the three levels of estimated VO_2_ at test and retest were compared for all subjects. All differences between the models were small and non-significant. The mean absolute and relative differences varied from 0.01 ± 0.08 to 0.02 ± 0.05 L/min (all n.s.) and 0.21 ± 6.89 to 1.42 ± 3.91% (all n.s.), respectively (Tables 9 and 10).

**Table 9:** Differences between HR–VO_2_ regression equations in model 1 and model 2 (means ± SD, and 95% confidence intervals (CI)) for all values

Participant	Differences between model 1 and model 2 with regard to the constituents (*y* intercepts and slope) and correlation outcomes (*r*)					
	Day 1			Day 2		
	*y* intercept	Slope	*r*	*y* intercept	Slope	*r*
Males						
1	0.23	− 0.0019	− 0.002	− 0.19	0.0016	0.000
2	0.42	− 0.0034	0.015	1.48	− 0.0118	0.002
3	− 0.78	0.0051	− 0.083	− 0.69	0.0051	0.014
4	0.58	− 0.0046	− 0.030	0.49	− 0.0036	0.031
5	0.75	− 0.0056	− 0.010	0.09	− 0.0006	0.008
6	− 0.16	0.0013	− 0.002	− 0.04	0.0003	0.000
7	0.59	− 0.0041	− 0.004	0.49	− 0.0036	0.006
Mean	0.23	− 0.0019	− 0.017	0.23	− 0.0018	0.000
SD	0.54	0.0038	0.032	0.69	0.0053	0.014
Females						
1	0.09	− 0.0008	0.002	0.44	− 0.0040	− 0.009
2	− 0.06	0.0004	0.002	0.02	− 0.0001	0.000
3	− 0.11	0.0009	− 0.001	− 0.15	0.0012	− 0.002
4	− 0.07	− 0.0006	0.000	− 0.11	0.0010	0.001
5	− 0.26	0.0023	− 0.007	− 0.12	0.0010	0.000
6	0.08	0.0006	0.003	0.15	− 0.0012	0.005
7	− 0.03	0.0002	0.004	− 0.09	0.0009	− 0.002
Mean	− 0.05	0.0004	0.000	0.02	− 0.0002	− 0.001
SD	0.12	0.0010	0.004	0.21	0.0019	0.004
All						
Mean	0.09	0.0007	− 0.008	0.13	− 0.0010	0.001
SD	0.40	0.0029	0.024	0.50	0.0039	0.010
* P* value	.412	.371	.216	.367	.367	.777
CI lower	− 0.14	− 0.002	− 0.02	− 0.16	− 0.003	− 0.01
CI upper	0.32	0.001	0.01	0.41	0.001	0.01

**Table 10:** Differences between model 1 and model 2 in the estimated VO_2_ based on three levels of HR-VO_2_ as well as absolute and relative differences between these outcomes (means ± SD, and 95% confidence intervals (CI)) for all values

Participant	Differences between model 1 and model 2 with regard to outcomes based on HR VO_2_ regression equations in estimations of VO_2_ based on these levels of HR, and the HR–VO_2_ regression equations at day 1 and day 2 and the absolute and relative differences between those outcomes														
	Lowest fifth of HR					Middle fifth of HR					Highest fifth of HR				
	HR F1	VO_2_:1	VO_2_:2	Abs diff	% diff	HR F3	VO_2_:1	VO_2_:2	Abs diff	% diff	HR F5	VO_2_:1	VO_2_:2	Abs diff	% diff
Males															
1	90	0.06	− 0.05	− 0.11	− 12.8	102	0.04	− 0.03	− 0.07	− 5.96	115	0.02	− 0.01	− 0.03	− 1.93
2	99	0.08	0.32	0.23	16.4	108	0.05	0.21	0.15	9.11	115	0.03	0.13	0.10	5.35
3	110	− 0.22	− 0.13	0.09	10.1	119	− 0.17	− 0.08	0.09	8.59	126	− 0.14	− 0.04	0.09	7.72
4	94	0.14	0.15	0.01	0.90	100	0.11	0.13	0.01	1.28	112	0.06	0.09	0.02	1.74
5	114	0.11	0.02	− 0.09	− 7.18	143	− 0.05	0.00	0.05	2.58	151	− 0.09	0.00	0.09	4.02
6	84	− 0.05	− 0.01	0.04	3.83	92	− 0.04	− 0.01	0.03	2.61	100	− 0.03	− 0.01	0.02	1.63
7	134	0.04	0.00	− 0.04	− 2.09	143	0.00	− 0.03	− 0.03	− 1.58	150	− 0.02	− 0.05	− 0.03	− 1.29
Mean	104	0.03	0.04	0.02	1.30	116	− 0.01	0.03	0.03	2.38	124	− 0.02	0.01	0.04	2.46
SD	17.2	0.12	0.15	0.12	9.95	20.7	0.09	0.10	0.08	5.33	19.5	0.07	0.07	0.06	3.49
Females															
1	107	0.00	0.01	0.01	0.78	116	0.00	− 0.02	− 0.02	− 1.34	122	− 0.01	− 0.05	− 0.04	− 2.43
2	97	− 0.02	0.01	0.03	5.24	104	− 0.02	0.01	0.03	3.83	116	− 0.02	0.01	0.02	2.37
3	119	− 0.01	− 0.01	0.00	− 0.01	130	0.00	0.01	0.00	0.22	140	0.01	0.02	0.01	0.39
4	102	− 0.01	− 0.01	0.00	− 0.34	113	0.00	0.00	0.00	0.02	125	0.01	0.01	0.01	0.33
5	97	− 0.04	− 0.02	0.01	1.28	107	− 0.01	− 0.01	0.00	− 0.16	128	0.03	0.01	− 0.03	− 1.94
6	102	0.02	0.02	0.01	1.02	117	0.01	0.00	0.00	− 0.12	124	0.00	0.00	− 0.01	− 0.42
7	97	− 0.01	− 0.01	0.00	− 0.14	111	0.00	0.01	0.01	0.75	143	0.00	0.04	0.03	1.88
Mean	103	− 0.01	0.00	0.01	1.12	114	0.00	0.00	0.00	0.46	128	0.00	0.00	0.00	0.02
SD	8.2	0.02	0.02	0.01	1.92	8.6	0.01	0.01	0.01	1.62	10.0	0.02	0.03	0.03	1.79
All															
Mean		0.01	0.02	0.01	1.21		− 0.01	0.01	0.02	1.42		− 0.01	0.01	0.02	1.24
SD		0.09	0.10	0.08	6.89		0.06	0.07	0.05	3.91		0.05	0.05	0.05	2.95
*P* value		.728	.466	.577	.522		.738	.529	.237	.198		.496	.511	.159	.139
CI lower		− 0.04	− 0.04	− 0.03	− 2.77		− 0.04	− 0.03	− 0.01	− 0.84		− 0.04	− 0.02	− 0.01	− 0.46
CI upper		0.06	0.08	0.06	5.19		0.03	0.05	0.05	3.68		0.02	0.04	0.05	2.94

## Discussion

An important feature of this study is that we have developed a transparent framework for analyses of the reproducibility of the HR method in laboratory conditions. It is characterized by positioning all HR values used in relation to both resting and maximal HR. This relates to both the HR–VO_2_ relations that were established in the laboratory, and the evaluation of them with three relevant HR levels that were obtained from walking commuting in field conditions. In this way, the relative localization of the measurement points of HRs used is clarified in a way that can be reproduced, and compared with future studies of these matters.

The main finding of this study is that there were no significant differences between test and retest in the constituents of the regression equations (*y* intercept, slope and *r* value) in model 1 and model 2. In line with that, there were no systematic differences in estimated absolute mean levels of VO_2_ between test and retest for either model. The relative differences between test and retest, based on estimations from three different levels of HR, were 1.91 ± 11.1, 1.71 ± 8.36 and 1.57 ± 7.34% (all n.s.) in model 1, and 3.12 ± 8.25, 3.13 ± 7.60 and 2.81 ± 7.47% (all n.s.) in model 2. However, some large individual differences were seen in both models, as indicated by the range of standard deviations for the relative differences (7.34–11.1%). Consequently, the 95% confidence intervals for the mean values of all subjects indicate variations of between about 8 and 14% for the three different estimations of relative differences in VO_2_ between test and retest. This spreading is further illustrated in the individual differences between test and retest, and the given 95% limits of agreements which are also illustrated in Figs. 2 and 3.

Another important finding was that there were no significant differences between models 1 and 2 in the constituents of the regression equations (*y* intercept, slope and *r* value) or in any of the outcome variables (estimated levels of oxygen uptake, or in the absolute or relative differences between test and retest).

An overall pattern of stability was discerned on the group levels between test and retest in VO_2_ and HR, which permits the present test–retest analyses of the outcomes of the HR–VO_2_ regression equations. The fact that we started the measurements with 15 min of rest in a supine position, and that all subjects were very physically active (cf. Table 1) can be relevant for this outcome. It should, at the same time, be kept in mind that habituation effects in HR with repeated measurements have been noted in studies of samples from the general population (Åstrand 1952; Åstrand 1976; Ekblom Bak et al. 2014). As a safeguard, a habituating pretest, as was applied by McCrory et al. (1997), is, therefore, recommended as a standard procedure.

A non-systematic test–retest variability in HR was also noted by McCrory et al. (1997). They even controlled for sex and some individual factors, such as being an emotional person, without being able to see any such relations. Berggren and Hohwü Christensen (1950) studied the HR–VO_2_ relation repeatedly in one person and found a variability in the heart rate of the same order of absolute magnitude in work rates demanding between 1 and 4 L of VO_2_. Thus, it is possible that this variability stands for an intrinsic feature of repeated HR–VO_2_ spot measures. And in many ways, this is reasonable since VO_2_, according to the Fick principle, is the product of heart rate, stroke volume and the difference between arterial and mixed venous oxygen content. Thus, levels of four different variables can, in principle, vary in response to a work rate, and still the resulting VO_2_ can be the same. From that perspective, it is not surprising that the HR may vary from time to time at a given exercise-induced VO_2_. It indicates that the biological steering mechanisms for these variables are not strictly controlled.

In individual cases, linearity between HR and VO_2_ has been indicated to sometimes end at near to maximal VO_2_ levels, with greater increases in VO_2max_ than in HR (Hohwü Christensen 1931; Davies 1968; Åstrand and Rodahl 1970, p 352, 414). Given that, it could be questionable to include values on maximal HR and VO_2_, as has been done in model 2 in our study, and thus it could be anticipated that the regression equations and outcomes of models 1 and 2 might differ. Including maximal HR and VO_2_ could, on the other hand, serve as an anchor, stabilizing effects of day to day variability of the regression equations that otherwise could come into play. One reason for such a role for HR values from maximal work rate is its low CV (Table 2) in comparison with those at the submaximal work rates. The fact that we did not see any significant differences between the outcomes in models 1 and 2 indicates the potential value of educational models that do not include measurements from maximal work rates. Furthermore, it indicates that research models for establishing the HR–VO_2_ relation may be adequate without maximal measurements. To include more submaximal measurements than the three that we have used, might, however, be a beneficial way to create even greater day to day stability in models based on only submaximal work rates, and deserve future studies.

One reason for the good reproducibility on the group level for model 1, despite only making use of three submaximal work rates, can be the span of the HR attained between work rates 1 and 3 (in average 37–56 beats per minute for males and females, respectively). It is important that the utilized range of HR from walking commuting is within the range of the HR attained from the three submaximal ergometer cycle work rates in the laboratory, which is the case for the females (cf. Tables 2 and 4). If instead VO_2_ were to be estimated from higher or lower HR than established from the submaximal work rates in the laboratory, it is possible that greater test–retest differences would have been observed (cf. Figure 1).

A comment on the field heart rates used is that the walking commuting HR were monitored in the morning and evening at slightly varying times. Given that the standardization criteria were followed, these times of HR collections are representative for walking commuting since the HR–VO_2_ relation during physical activity is not affected by the time of the day (McCrory et al. 1997).

Another comment favoring a stability in the measurement conditions is that the mean values for the positions of per cent HR_max_ used to establish the HR–VO_2_ relations related well to the expected VO_2_ relative to VO_2max_ in both sexes (Table 3) (Londoree and Ames 1976).

Our results are in line with those of McCrory et al. (1997), and considerably more favorable in relation to using the HR–VO_2_ method than those indicated by Christensen et al. (1983). There are several explanations for that. The measures used by Christensen et al. (1983) for establishing HR–VO_2_ regression equations were resting and sitting, as well as three low to intermediate exercise rates on an ergometer cycle (8–100 W) and three exercise rates on a treadmill, thus altogether eight measurement points. For both the slope and the *y* intercept of the regression equations, the measurements at low levels of HR are, under those circumstances, more influential. At the same time, it is well known that the HR–VO_2_ ratios at rest and sitting are quite unstable, resulting in variations in regression equations (Booyens and Hervey 1960; Montoye et al. 1996, p. 103; McCrory et al. 1997). Between very low intensities of exercise and rest, the slope of the HR–VO_2_ linear relationship will have a different angle compared to higher intensities (Achten and Jeukendrup 2003; Booyens and Harvey 1960; Luke et al. 1997; Spurr et al. 1988), which could be another reason for the results of Christensen et al. (1983). Furthermore, mixing the work forms on cycle ergometer and treadmill as the bases for the HR–VO_2_ measures is in itself problematic since HR response for a given VO_2_ differs in these different forms of movement (Lafortuna et al. 2008). This creates a greater risk for non-stability in the regression equations with repeated measurements. Finally, the measures of 24 h HR by Christensen et al. (1983) resulted in a mean value of 86 beats per minute. In line with the reasoning in the Introduction (cf. Fig. 1), a HR close to the endpoint of the spectrum of measurement points forming the regression equation will with greater plausibility lead to a lower reproducibility. Another potential explanation for their results relates to their use of a heterogeneous sample of predominantly patients and large variations in age, whereas we studied a sample of healthy and physically active middle-aged individuals. Having stated that, one has to keep in mind that the external validity of our findings in relation to other types of participants is uncertain. Thus, to forward the general knowledge in these respects, there is indeed a need for further studies of these matters.

As stated in “Introduction”, the studied models 1 and 2 mimic those that have been used at our research and educational setting. In our mind, it would be of great value to further the understanding of if the HR method can be optimized through using more submaximal measuring points when establishing HR–VO_2_ relation in laboratory. Berggren and Hohwü-Christensen (1950) undertook a number of HR and VO_2_ measurements on one individual, and their results indicate a variation in oxygen pulse for a given oxygen uptake. However, we do not know if such variations occur “within a day”, or are the result of “between-day variations”. Independent of the cause for them, using, e.g., five or seven submaximal measurements points might stabilize the day to day variation, as compared to using only three points, and systematic studies of this deserve to be undertaken.

In approaching the end of this discussion, it is reasonable to also point at the fact that we do not know anything about the external validity of the HR method in the laboratory in relation to field conditions such as during walking commuting. Two studies have looked at the intensity of walking commuting using different HR methods (Kokkonen-Harjula et al. 1988; Oja et al. 1991). However, none of these studies considered that, for reasons such as cardiovascular drift with prolonged work durations (Achten and Jeukendrup 2003; Coyle and Gonzalez-Alonso 2001; Saltin and Stenberg 1964) or stress due to traffic conditions (Carroll et al. 2009; Lambiase et al. 2012), the relationship measured in the laboratory may differ when being in a walking commuting environment, and that consequently the indicated intensity of walking commuting might be incorrect. This will be the focus in our further studies.

We have, as pointed out in the beginning of “Discussion”, developed a framework for studying these matters in terms of relating all HRs used to the maximal HR (%maxHR) and the relative position of the HR in between the resting and the max HR (%HRR). In future studies, we do also suggest that the body temperature is monitored since this factor influences the metabolism and may affect the blood flow distribution and thereby also the constituents of the Fick principle, with possible effects on HR–VO_2_ relations.

In conclusion, the present study demonstrates good reproducibility on the group level of HR–VO_2_ relations established through cycle ergometer exercise with healthy and physically active middle-aged walking commuters as participants in laboratory conditions, and evaluated at three levels of heart rates from walking commuting that represent moderate exercise intensities.

## Abbreviations

CI: Confidence interval
CV: Coefficient of variation
PACS Q1: Physically active commuting in Greater Stockholm, Questionnaire 1
HR: Heart rate
N: Newton
RPE: Rated perceived exertion
SD: Standard deviation
VO_2_: Volume of oxygen uptake
W: Watt
